# Evolutionary Elongation of the Time Window of Integration in Auditory Cortex: Macaque vs. Human Comparison of the Effects of Sound Duration on Auditory Evoked Potentials

**DOI:** 10.3389/fnins.2019.00630

**Published:** 2019-06-24

**Authors:** Kosuke Itoh, Masafumi Nejime, Naho Konoike, Katsuki Nakamura, Tsutomu Nakada

**Affiliations:** ^1^Center for Integrated Human Brain Science, Brain Research Institute, Niigata University, Niigata, Japan; ^2^Cognitive Neuroscience Section, Primate Research Institute, Kyoto University, Kyoto, Japan

**Keywords:** event-related potential, temporal integration, auditory late latency response, non-human primate, evolution

## Abstract

The auditory cortex integrates auditory information over time to obtain neural representations of sound events, the time scale of which critically affects perception. This work investigated the species differences in the time scale of integration by comparing humans and monkeys regarding how their scalp-recorded cortical auditory evoked potentials (CAEPs) decrease in amplitude as stimulus duration is shortened from 100 ms (or longer) to 2 ms. Cortical circuits tuned to processing sounds at short time scales would continue to produce large CAEPs to brief sounds whereas those tuned to longer time scales would produce diminished responses. Four peaks were identified in the CAEPs and labeled P1, N1, P2, and N2 in humans and mP1, mN1, mP2, and mN2 in monkeys. In humans, the N1 diminished in amplitude as sound duration was decreased, consistent with the previously described temporal integration window of N1 (>50 ms). In macaques, by contrast, the mN1 was unaffected by sound duration, and it was clearly elicited by even the briefest sounds. Brief sounds also elicited significant mN2 in the macaque, but not the human N2. Regarding earlier latencies, both P1 (humans) and mP1 (macaques) were elicited at their full amplitudes even by the briefest sounds. These findings suggest an elongation of the time scale of late stages of human auditory cortical processing, as reflected by N1/mN1 and later CAEP components. Longer time scales of integration would allow neural representations of complex auditory features that characterize speech and music.

## Introduction

Auditory information is integrated over time for obtaining neural representations of auditory events in the brain (Heil and Neubauer, 2001; Lerner et al., 2011; Farbood et al., 2015). The time scale of integration is a critical parameter of auditory processing that determines how sounds are represented in the brain and in perception (Moore, 2003; Sussman, 2005; Lerner et al., 2011, 2014; Farbood et al., 2015). For example, the perceptual distinction between voiced and unvoiced speech sounds (e.g., /ba/ vs. /pa/) depends on whether the voice onset interval is longer or shorter than about 30 ms, respectively. A necessary condition for such perception to be possible is that the time scale for syllable perception is sufficiently longer than the voice onset interval.

Temporal integration occurs at each level of auditory processing, from peripheral to central, and the time scale of neural representations of continuously incoming sounds generally becomes longer at higher levels of sensory processing, reflecting the accumulation of information over time (Lerner et al., 2011; Hasson et al., 2015). Regarding the final stages of auditory processing that are directly relevant to the perception and cognition of sounds, the time scale of auditory cortical functions is implicitly assumed to be similar across human and non-human primate species. This assumption, however, has never been explicitly tested to our knowledge. It is worthwhile to examine the possibility that the time scale of auditory cortical processing has extended over the course of primate brain evolution to enable representations of complex sound features, such as those that characterize speech and music. In fact, the macaque homologs of human cortical auditory evoked potentials (CAEPs) have shorter latencies compared with the human CAEP (Fishman et al., 2000b; Itoh et al., 2015), which strongly suggests shorter time scales of auditory cortical processing in the macaque cortex.

This work investigates species differences in the time scale of auditory cortical processing by comparing how the N1 and other components of human and macaque CAEP diminish in amplitude as the sound duration is decreased from 100 ms or longer to 2 ms. A long time scale of integration entails that CAEP amplitudes become small as sound duration is decreased, while neural circuits tuned to processing auditory signals at short times scales would continue to produce large CAEP responses to brief stimuli.

## Materials and Methods

### Subjects

Animal experiments were carried out on four young adult rhesus monkeys (*Macaca mulatta*; two males and two females, 4–5 kg, 4–7 years old). These animals displayed no behavioral signs of hearing deficits. The animals were not restricted from food and water throughout experimental period. The Animal Care and Use Committee of Kyoto University approved the study, and all experiments were performed in accordance with the Guide for the Care and Use of Laboratory Animals of the National Research Council (1996) and the Guide for Care and Use of Laboratory Primates of Kyoto University. All experiments were conducted at Kyoto University.

Human experiments were carried out on twelve, audiologically normal, right-handed volunteers (18–23 years old, two males). All volunteers provided written informed consent for participation prior to enrollment. Human experiments were conducted at the University of Niigata after the Internal Review Board of the University of Niigata approved the study. All experiments were performed in accordance with the Declaration of Helsinki.

### Stimuli and Apparatus

The stimuli, apparatus, and procedure used were identical across the macaque and human experiments unless otherwise noted.

The auditory stimulus was a pure tone (1500 Hz) with variable durations of 200 (human experiment only), 100, 50, 10, 5, 3, or 2 ms with a linear rise/fall time of 1 ms. The stimulus duration contained the rise and fall times. The use of the 200 ms stimulus was unnecessary in the macaque experiment, because all the macaque CAEP components occurred before 200 ms in latency, meaning that no further temporal integration would occur for this stimulus.

These sounds were presented in a randomized sequence with a variable interstimulus interval (ISI) of 300–400 ms. The distribution of the ISI within this interval was uniform. A total of 1400 trials in the monkey experiments and 600 in the human experiments were conducted for each variable duration condition. Sounds were digitally synthesized with Adobe Audition CS6, saved as wave files (16 bit, 48 kHz), and played back on a computer using Presentation software (Neurobehavioral systems, Berkeley, CA) and Sound Blaster audio hardware (Creative Technology, Jurong East, Singapore). Sound playback latencies with respect to the trigger signal were measured and corrected in post-processing. Sounds were presented using a loud speaker (MSP7 Studio; Yamaha, Hamamatsu, Japan) placed 0.6 m from the monkey’s head, or 1.2 m from the human’s head. The speaker was placed straight ahead of the subject for both species, and sound intensity was in the range of 65–70 dB SPL as measured at the position of the head.

In the animal experiments, the monkeys sat in a primate chair and passively listened to the sounds in a sound-attenuated room. Occasional rest phases were provided (approximately once every 20 min) to check the state of the animal, provide food and/or water, and/or to maintain electrode impedance. Polystyrene blocks placed on the left and right sides of the monkey’s head were used to restrict left-right rotational head movements. Horizontal bars placed above the nose and supraorbital ridge restricted upward rotations and forward movements. The detailed protocol of animal preparation has been described elsewhere (Itoh et al., 2015). In the human experiments, participants sat in a comfortable chair in a sound-attenuated room and listened passively to the sounds without being given specific instructions.

### CAEP Recording and Analyses (Macaques)

Silver electrodes used for human sleep recordings (NE-136A, Nihon Kohden, Japan) were placed on the monkey’s scalp and earlobes according to the International 10–20 system to record the electroencephalogram (EEG). An electrooculogram (EOG) was also recorded using an electrode placed to the lower left of the left eye to monitor ocular artifacts. After the electrodes were placed using collodion, electrode gel was applied to lower the impedance to below 5 kΩ. Twelve electrodes were applied (F3, F4, C3, C4, P3, P4, Fz, Cz, Pz, A1, A2, and EOG). Because there was no clear or consistent left-right asymmetry, analyses were performed using only the midline electrodes (Fz, Cz, and Pz) where the CAEP amplitudes were maximal. All EEG and EOG channels were referenced to Cz during the recordings and re-referenced to the linked earlobes (i.e., the average of A1 and A2) during post-processing. The EEG and EOG were amplified (16 bit, 0.1 μV/LSB precision), bandpass filtered (0.016–250 Hz), and sampled at 1000 Hz using BrainAmp MR plus (Brain Products, Munich, Germany).

**FIGURE 1:**
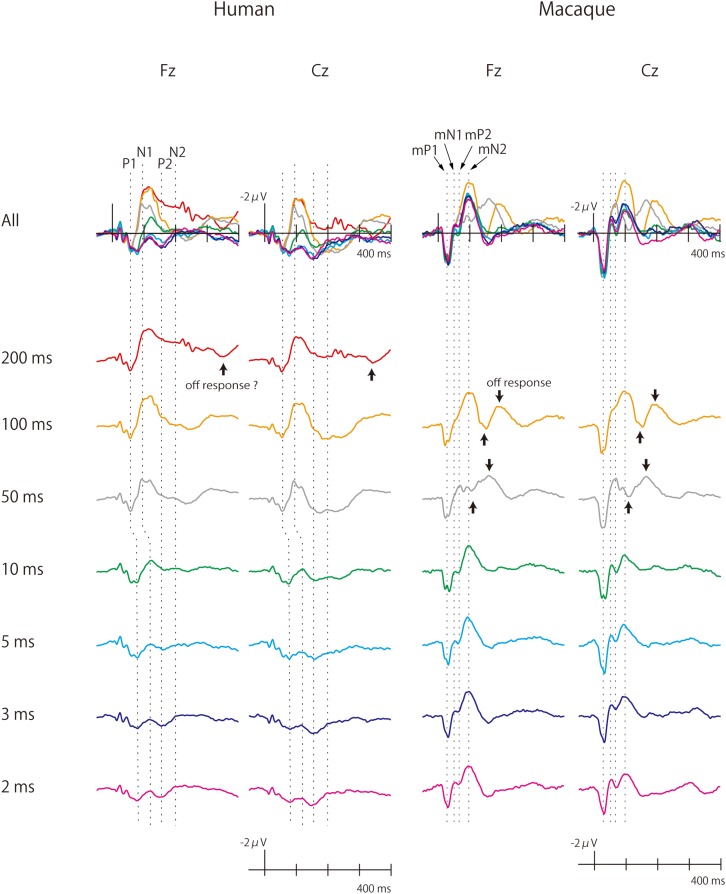
Human and monkey scalp-recorded CAEPs elicited by pure-tone stimuli of different durations. The macaque cortex continued to produce large CAEPs as sound duration was shortened. Off-responses were observed more clearly in monkeys.

In post-processing, the data were bandpass filtered (1–100 Hz, 12 dB/oct), segmented, time-locked to the onset of the stimulus (−50 to 400 ms), adjusted to the baseline using the prestimulus-period average, artifact filtered (±150 μV relative to baseline), and then averaged to obtain the CAEPs. The number of non-rejected time segments was 1198–1231 (86–88%), 1194–1218 (85–87%), 1312–1372 (94–98%), and 1304–1317 (93–94%), respectively, for the four monkeys. EMSE Suite version 5.5.1 (Source Signal Imaging, La Mesa, CA, United States) was used for the processing of electrophysiological data. The amplitude of mP1 was represented by the average in the time range 20–40 ms at Cz, while the time window for mN1 was 40–60 ms at Cz and that for mN2 was 90–110 ms at Fz or Cz. These time slots and electrodes were determined by examining the group-averaged CAEP waveforms (Figure 1). For each component, the time window was centered at the peak latency, and the electrode of analysis represented the spatial peak of the response in the 2 ms condition.

### CAEP Recording and Analyses (Humans)

Electroencephalograms were recorded from five silver electrodes placed along the midline (Fpz, Fz, Cz, Pz, and Oz) and the left and right earlobes (A1 and A2) according to the International 10–20 system. Horizontal and vertical EOGs (HEOG and VEOG) were recorded from the left eye. The EEG and EOG were amplified (16 bit, 0.1 μV/LSB precision), bandpass filtered (0.016–250 Hz), and sampled at 1000 Hz using BrainAmp MR plus (Brain Products, Munich, Germany). The data were re-referenced offline to the linked ears.

The procedures for obtaining the human CAEPs were identical to those used in the animal experiments, except that the criteria for artifact rejection was ± 100 μV. The number of non-rejected epochs was in the range of 351–571 (59–95%) with a mean of 508 (85%). The time windows and electrodes chosen to evaluate the CAEP amplitudes were 45–65 ms at Cz, respectively, for P1. Because the peak latency of N1 changed noticeably with sound duration, the time window for the N1 analyses was 80–110 ms for the 50 ms and 100 ms conditions and 105–135 ms for the other conditions, all at the Fz electrode. The amplitude of P1-N1 deflection was also calculated, by subtracting the P1 amplitude at Fz from the N1 amplitude at Fz. For the N2 component, the time window was 150–250 ms at Fz. These time slots and electrodes were determined by examining the group-averaged CAEP waveforms (Figure 1). For each component, the time window was centered at the peak latency, and the electrode represented the spatial peak of the response in the 2 ms condition.

### Species Comparisons

We analyzed and interpreted our results per the hypothesis that the human P1-N1-P2- N2 complex corresponds, peak-to-peak, to the macaque mP1-mN1-mP2-mN2 complex (Fishman et al., 2000b, 2014; Fishman and Steinschneider, 2012; Itoh et al., 2015). Rationales for this assumption are presented in the Discussion section.

Considering the latency of N1 and mN1, which were approximately 100 ms or shorter, the amplitudes of these waves in the 100 ms condition represented their (nearly) maximum amplitude (for the present ISI) because further temporal integration could not contribute to an amplitude increase. For the same reason, the P1/mP1 amplitudes in the 50 and 100 ms conditions, the N2 amplitude in the 200 ms condition, and the mN2 amplitude in the 100 ms condition were also representative of their maximum amplitudes These situations allowed us to quantitatively analyze how P1/mP1, N1/mN1, and N2/mN2 diminished in amplitude from their maximum values as sound duration decreased.

Species differences for the effects of stimulus duration on P1/mP1, N1/mN1, and N2/mN2 were statistically evaluated using a mixed linear model using a random intercept and slope for each subject and a variance component covariance structure and maximum likelihood estimation. Each data sample in the analysis represented one epoch of non-rejected EEG recording data. Species (Human/Macaque) was a between-subject factor, and Duration (2/3/5/10/50/100 ms in the Macaque, and 2/3/5/10/50/100/200 ms in the Human) was a within-subject factor. That is, the regression equation included the design matrix that modeled the fixed effects of Species, Duration, and their interaction, as well as the design matrix for the random effects of Duration that modeled random intercepts and slopes at the subject level. Pairwise comparisons between the stimuli conditions were performed in each species to reveal the pattern of amplitude change across different stimulus durations. These results were adjusted for multiple comparisons by using the method of Šidák (1967). Statistical analyses were performed using IBM SPSS Statistics version 22 (IBM, Armonk, NY, United States).

## Results

### Overview

Grand-averaged waveforms of the human and macaque CAEPs to pure-tone stimuli of different durations are shown in Figure 1. As a general finding, the human and macaque CAEPs had the same number of peaks with matched polarities, although the latencies were overall shorter in the macaque. The P1 (55 ms, peak latency), N1 (90–130 ms), P2 (160 ms), and N2 (200 ms) waves constituted the human CAEP. The peak latencies of P1 and N1 were longer for brief (≤10 ms) sounds than for long sounds. The N2 response was weak and difficult to identify in most conditions. The macaque CAEP also comprised a series of four transient responses, which were labeled as macaque P1 (mP1, 30 ms), macaque N1 (mN1, 55 ms), macaque P2 (mP2, 70 ms) and macaque N2 (mN2, 95 ms), to indicate their polarity and order (Itoh et al., 2015). In addition, off responses, or positive-negative deflections that were time-locked to sound offset, were unambiguously elicited by the 50 ms and 100 ms stimuli in monkeys, while such responses were not clearly identified in the human CAEP (Figure 1).

Sound duration affected the CAEP waveforms in both humans and macaques, but in different manners (Figure 1). In humans, the auditory cortical responses diminished in amplitude as sound duration was shortened, and waves later than P1 were not clearly elicited by stimuli shorter than 10 ms. In monkeys, by contrast, substantial cortical responses remained even to the briefest sound, and the full complex of mP1-mN1-mP2-mN2 peaks was clearly identified in the 2 ms condition. These observations were supported statistically as described below.

### Effects of Sound Duration

#### P1 and mP1

**FIGURE 2:**
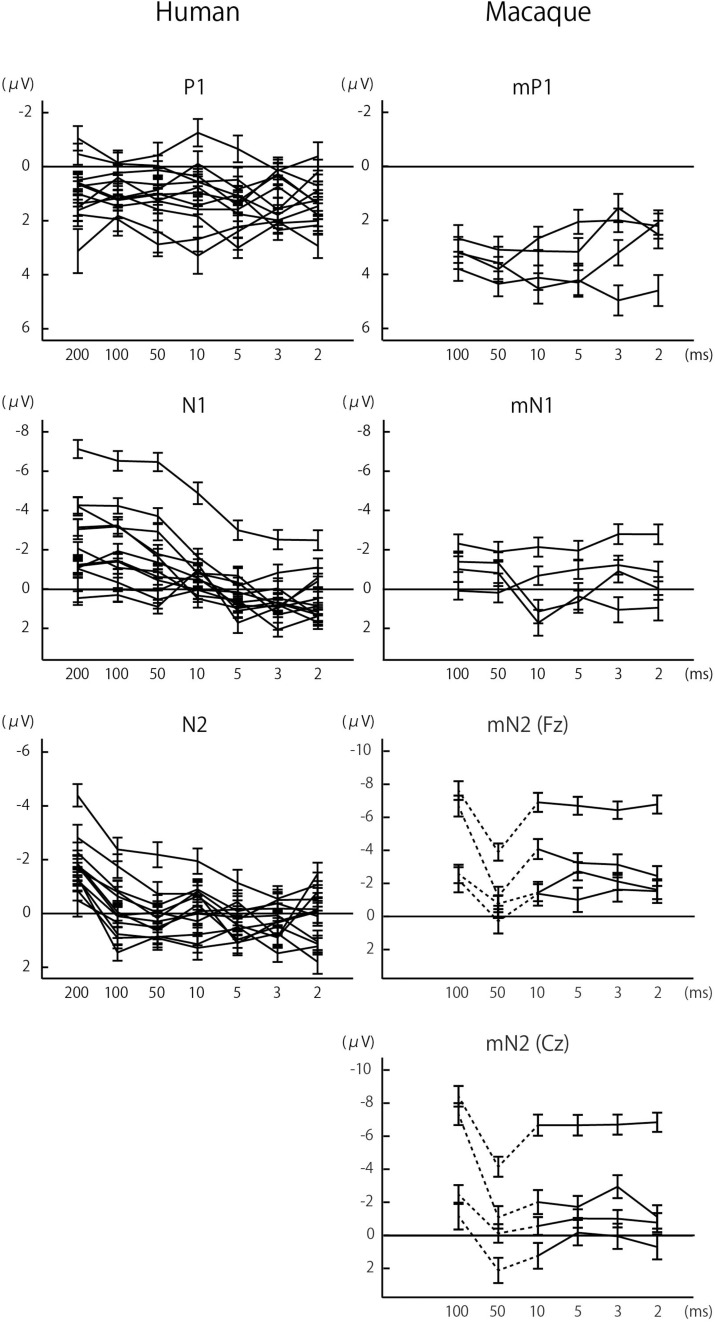
Effects of sound duration on the amplitudes of P1/mP1, N1/mN1, and N2/mN2. The amplitudes of human N1 and N2 significantly decreased with sound duration, *p* < 0.05 (Table 1), while such effect was less evident with other components. Each line represents a single subject, and the error bars represent standard errors.

In both humans and macaques, a sound duration of as short as 2 ms was sufficient to elicit significant P1/mP1 responses (Figure 1). The amplitudes of P1 and mP1 were significantly different from the baseline of zero microvolt in all stimulus conditions, including the 2 ms condition (Table 1). Furthermore, the P1 and mP1 amplitudes did not increase with the duration of the stimulus (Figure 2). The mixed linear model analysis revealed that the effect of Duration was not significant, *F*(6,91.0) = 1.2, *p* = 0.311, and the Species × Duration interaction was significant, *F*(5,82.0) = 2.4, *p* = 0.048. When analyzed separately in each species, the effect of Duration was not significant in humans, *F*(6,69.8) = 1.7, *p* = 0.136, nor in monkeys, *F*(5,19.7) = 1.3, *p* = 0.294. These results indicated that the time window of integration was quite short (<2 ms) for P1 and mP1.

**TABLE 1:** CAEP amplitudes.

**Duration (ms)**	**Humans**			**Macaques**			
	**P1**	**N1**	**N2**	**mP1**	**mN1**	**mN2 (Fz)**	**mN2 (Cz)**
2	1.3${}^{†}$ [0.8, 1.9]	0.4^*^ [−0.6, 1.3]	0.1^*^ [−0.4, 0.6]	2.8${}^{†}$ [1.8, 3,8]	−0.7 [−2.1, 0.7]	−3.1^*^${}^{†}$ [−5.8, −0.4]	−2.0^*^ [−5.6, 1.6]
3	1.2${}^{†}$ [0.7, 1.8]	0.4^*^ [−0.5, 1.4]	0.3^*^ [−0.2, 0.8]	2.9${}^{†}$ [1.9, 3.9]	−1.0 [−2.4, 0.4]	−3.3${}^{†}$ [−6.0, −0.6]	−2.6^*^ [−6.3, 0.9]
5	1.4${}^{†}$ [0.8, 2.0]	0.2^*^ [−0.8, 1.1]	0.3^*^ [−0.2, 0.8]	3.4${}^{†}$ [2.5, 4.4]	−0.5 [−1.9, 0.9]	−3.4${}^{†}$ [−6.1, −0.7]	−2.4^*^ [−6.0, 1.2]
10	1.1${}^{†}$ [0.5, 1.6]	−0.9^*^ [−1.8, 0.1]	−0.2^*^ [−0.7, 0.3]	3.6${}^{†}$ [2.6, 4.6]	−0.0 [−1.4, 1.3]	−3.4${}^{†}$ [−6.2, −0.7]	−2.0^*^ [−5.6, 1.6]
50	1.0${}^{†}$ [0.5, 1.6]	−1.5${}^{†}$ [−2.5, −0.6]	0.1^*^ [−0.4, 0.6]	3.7${}^{†}$ [2.8, 4.7]	−1.0 [−2.3, 0.4]	−1.4 [−5.1, 1.3]	−0.8^*^ [−4.4, 2.8]
100	0.9${}^{†}$ [0.3, 1.4]	−2.2${}^{†}$ [−3.1, −1.2]	−0.2^*^ [−0.7, 0.3]	3.2${}^{†}$ [2.3, 4.1]	−1.0 [−2.5, 0.2]	−4.7${}^{†}$ [−7.5, −2.0]	−4.8${}^{†}$ [−8.4, −1.2]
200	1.0${}^{†}$ [0.4, 1.5]	−2.3${}^{†}$ [−3.3, −1.4]	−1.8${}^{†}$ [−2.3, −1.3]	N.A.	N.A.	N.A.	N.A.

The values indicate the mean and the 95% confidence interval in μV. Asterisks (^*^) denote that the amplitude was significantly smaller compared to the 100 ms condition or the 200 ms condition, *P* < 0.05 (Šidák-corrected). Daggers *(${}^{†}$)* denote that the amplitude was significantly different from the baseline as determined by the 95% confidence interval. N.A.: data not available.

**FIGURE 3:**
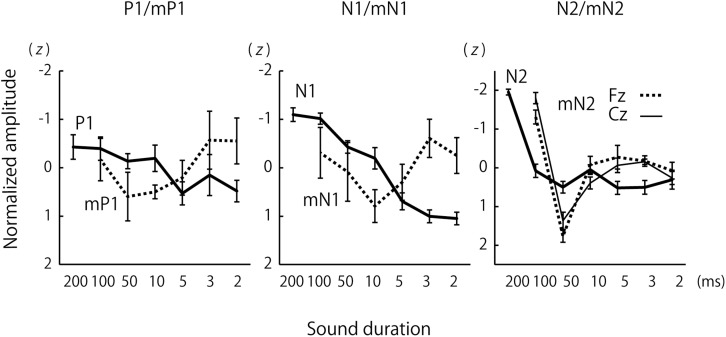
Group comparisons of the effects of sound duration on the amplitudes of P1/mP1, N1/mN1, and N2/mN2. The lines represent group-averages that were calculated after the CAEP amplitudes were normalized to *z*-scores across the sound duration conditions for each subject. The error bars represent standard errors.

For a more direct species comparison of the effects of sound duration on the P1 and mP1 as well as other CAEP components, group-averaged CAEP amplitudes were obtained for each species after the amplitudes were normalized to *z*-scores across the stimulus conditions for each subject (Figure 3). The results confirmed the small effects of sound duration on the mP1 and P1 amplitudes. Although the human P1 amplitude appeared to decrease slightly with sound duration, this could be explained by an overlap of the rising phase of N1 that significantly increased in amplitude with sound duration (see the next section).

#### N1 and mN1

We observed a significant species difference in the effects of sound duration on the mN1 and N1 amplitudes (Figures 2, 3). The Species × Duration interaction was significant, *F*(5,67.4) = 6.8, *p* < 0.001, and, when tested separately in each group, the effect of Duration was significant in humans, *F*(6,71.6) = 27.2, *p* < 0.001, but not in monkeys, *F*(5,19.8) = 1.3, *p* = 0.306. In humans, pairwise comparisons indicated that the N1 amplitudes in the 2, 3, 5, and 10 ms conditions were all significantly smaller compared to 100 ms or longer stimuli, *p* < 0.05 (Šidák -corrected) (Table 1). In monkeys, none of the pairwise comparisons were significant, *p* > 0.05 (Šidák-corrected) (Table 1).

#### N2 and Frontal mN2

The analysis of mN2 and N2 amplitudes was made difficult by the fact that a substantial off-response overlapped the macaque mN2 when the sound duration was 50 ms (Figure 1). The sound offset evoked a positive wave at a latency of 50 ms (Figure 1), resulting in an apparent decrease in the amplitude of the onset-evoked mN2 when the sound duration was 50 ms (Figures 1–3). Therefore, the results were interpreted with caution regarding the 50 ms condition in monkeys.

We first analyzed the mN2 measured at the Fz electrode, referred to here as the frontal mN2. The rationale for this choice of electrode was due to the greatest mN2 amplitude measured at Fz, except for the 100 ms condition (Figure 1 and Table 1).

The regression analysis revealed that the Species × Duration interaction was significant, *F*(5,84.5) = 10.0, *p* < 0.001, indicating that the frontal mN2 and N2 were affected differently by sound duration. When tested separately in each species, the effect of Duration was significant in humans, *F*(6,69.7) = 22.5, *p* < 0.001, and pairwise comparisons revealed that the N2 amplitude was greater for the 200 ms stimulus compared to all other conditions, *p* < 0.05 (Šidák-corrected) (Table 1). The effect of Duration was also significant in monkeys, *F*(5,19.4) = 11.7, *p* < 0.001. In the *post-hoc* analysis, all pairwise comparisons with the 50 ms condition were significant, which were explained by the overlap of the off-response. More importantly, there was a significant difference in the frontal mN2 amplitude between the 100 ms and the 2 ms conditions, *p* < 0.05 (Šidák-corrected), but not in any other pairwise comparisons (Table 1).

Similar results were obtained when the entire analysis was performed after excluding the data for the 50 ms condition in monkeys. The Species × Duration interaction was significant, *F*(4,74.9) = 2.8, *p* = 0.030, and the effect of Duration was significant in humans (as shown above) and in monkeys, *F*(4,15.2) = 4.2, *p* = 0.017. In the macaque, pairwise comparisons again revealed a significant difference in the frontal mN2 amplitude between the 100 ms and 2 ms conditions, *p* < 0.05 (Šidák-corrected), but not in any other pairwise comparisons.

Importantly, the frontal mN2 amplitude was significantly different from the baseline of zero microvolt in all conditions except the 50 ms condition, as determined by the 95% confidence interval (Table 1): this was true even for the briefest stimulus. Individual monkeys’ response profiles confirm significant frontal mN2 responses to brief sounds (Figure 2), and the CAEP waveforms also show clear frontal mN2 responses to these stimuli (Figure 1). These results indicated that a few ms of temporal integration was sufficient to elicit a significant frontal mN2 in monkeys.

In humans, by contrast, the N2 amplitude significantly diminished as the sound duration was shortened from 200 to 100 ms (Figures 1, 2), and the amplitude was not significantly different from the basline in all conditions except the 200 ms condition, as determined by the 95% confidence interval of the estimates of the N2 amplitude (Table 1). These results indicated that the time window of integration for the N2 was longer than 100 ms in humans.

#### Central mN2

The waveforms in Figure 1 indicate a large mN2-like response elicited by the 100 ms stimulus at the Cz electrode, although the mN2 amplitude was always greatest at Fz in all other conditions. In general, a difference in scalp distribution indicates different neural sources. Therefore, whether the Cz-maximal mN2-like response in the 100 ms condition was generated by the same neural sources as those that generated the Fz-maximal mN2 response in other stimulus conditions was questionable.

One possible explanation was that, as the stimulus duration increased, a novel CAEP component with a central distribution began to overlap the genuine mN2 that had a frontal distribution. Another equally valid interpretation was to consider mN2 as a composite wave that had multiple generators: one frontal subcomponent maximal at Fz and another central subcomponent at Cz. This is a matter of definition, and we have decided to take this latter view.

To examine how the central subcomponent of mN2, or central mN2, was affected by sound duration, the statistical analyses in section N2 and Frontal mN2 were repeated using the Cz electrode. The regression analysis revealed that the Species × Duration interaction was significant, *F*(5,88.2) = 12.2, *p* < 0.001, indicating that the central mN2 and N2 were affected differently by sound duration. When tested separately in each species, the effect of Duration was significant in humans (see Section 3.2.3) and in monkeys, *F*(5,19.3) = 13.3, *p* < 0.001. In the *post-hoc* analysis of central mN2, all pairwise comparisons with the 100 ms condition were significant, *p* < 0.05 (Šidák-corrected) (Table 1).

Similar results were obtained when the entire analysis was performed after excluding the data for the 50 ms condition in monkeys. The Species × Duration interaction was significant, *F*(4,74.9) = 9.4, *p* < 0.001, and the effect of Duration was significant in humans (as shown above) and in monkeys, *F*(4,15.2) = 8.9, *p* < 0.001. In the macaque, pairwise comparisons revealed a significant difference in the central mN2 amplitude between the 100 ms and all other conditions, *p* < 0.05 (Šidák-corrected).

These results suggested that the integration window for the central mN2 was between 50 and 100 ms, whereas that for the human N2 was between 100 and 200 ms. In contrast to the frontal mN2, which was elicited by the briefest stimulus, the central mN2 amplitude at Cz was not significantly different from zero for sounds shorter than 100 ms, as determined by the 95% confidence interval (Table 1).

## Discussion

Temporal integration is a fundamental principle of neural processing, and the time scale of integration is one of the most important parameters of brain function. Analogous to how a large receptive field in vision allows neural representations of complex figures that extend over space, a long time scale in auditory processing enables representations of complex auditory features due to accumulation of information over time. This work provides the first experimental evidence, to our knowledge, that the human brain has a longer time scale than the macaque brain regarding late stages of auditory cortical processing as indexed by N1 and later components of CAEP.

The human N1 significantly decreased in amplitude as sound duration was shortened from 100 ms to 2 ms. This is consistent with the previously described temporal integration window of N1, which is 50 ms or longer (Onishi and Davis, 1968; Alain et al., 1997). While the temporal integration window of macaque mN1 is unknown, its latency (55 ms) constrains it to be shorter than the temporal integration window of human N1. Our experiment provided empirical proof of this notion by demonstrating that substantial mN1 responses persist for brief stimuli that are too short to elicit a clear N1 in humans.

Further evidence for a shorter time scale of auditory processing in monkeys was obtained by CAEP components elicited after N1 in latency. A clear frontal mN2 response was elicited by very brief sounds (<10 ms) at the Fz electrode in monkeys, indicating a short time window of temporal integration for the frontal subcomponent of mN2. The central subcomponent of mN2 measured at Cz had a longer time window of integration (50–100 ms), but it was shorter than that of the human N2 (>100 ms). These contrasting results regarding the frontal and the central subcomponents of mN2 could be interpreted in two ways. First, it was possible that a common set of neurons that generated both the frontal mN2 and central mN2 had a relatively long time window of integration (>50 ms), but that it was also activated by very short stimuli; that is, there were two stages of temporal integration, one occurring early, and another occurring late. Second, it was also possible that the frontal mN2 and the central mN2 reflected the neural activities of different ensembles of neurons, as suggested by the difference in their scalp distributions. In this case, it would be reasonable to conclude that the frontal mN2 and the central mN2 had different time windows of integration, albeit the similarity in peak latency. Although there was an abrupt increase in the mN2 amplitude at Fz between the 50 ms and 100 ms conditions, this could be interpreted as an overlap of the central mN2 rather than an increase in the frontal mN2 amplitude itself.

In addition, robust off-responses were elicited by long stimuli (50 and 100 ms) in monkeys, while such waves were difficult to identify in humans. The latter result is consistent with previous findings that off-responses in humans are typically elicited with sounds longer than 100 ms (Hillyard and Picton, 1978; Pantev et al., 1996). To the best our knowledge, this is the first report to describe scalp-recorded auditory off-responses in the macaque monkey.

We do not assert that the absence of N1/mN1 and later CAEP responses to brief sounds signified that the brief stimuli were undetected in the auditory cortex. These sounds, in fact, evoked clear mP1/P1 in both humans and monkeys. Because the temporal integration windows of P1 and mP1 must be shorter than their latencies (50 and 30 ms, respectively), their amplitudes in the 50 and 100 ms conditions represented their saturated maxima. As shortening the sound duration to 2 ms did not result in any significant decrease in amplitude from these maxima, the time scale of auditory processing at an initial stage of cortical processing as indexed by P1/mP1 was quite short (<2 ms), both in humans and monkeys. Our P1 finding in the human participants confirm, and also extends, an earlier magnetoencephalographic observation that the magnetic counterpart of P1 (or P1m) was barely affected by the duration of sound in the range of 34–76 ms (Ross et al., 2009).

Importantly, the above discussions are based on the premise that the N1 and mN1 represent functional homologs of each other. Establishing a cross-species correspondence of evoked potential components is a difficult issue. However, two lines of evidence support the assumption of N1-mN1 correspondence: morphological and functional. First, there is a one-to-one correspondence in the morphological structure of human and macaque CAEPs, when they are recorded over the scalp. Both the human and macaque scalp-recorded CAEP comprise four transient responses followed by a sustained potential, whose polarities are completely matched (Itoh et al., 2015), and the simplest interpretation would be that the human P1-N1-P2-N2-SP peaks correspond to mP1-mN1-mP2-mN2-mSP peaks in the macaque, respectively. The latencies of the peaks are overall shorter in the macaque, but it is a general property of the macaque event-related potentials (Woodman, 2012). Considering that the macaque auditory evoked responses already have shorter latencies at the level of brainstem (Legatt et al., 1986), the fact that the CAEP latencies are short in the macaque in fact supports, rather than argues against, the proposed correspondence.

Second, and more important, mN1 represents a stage of auditory processing that is functionally comparable to that represented by the human N1. In humans, a contextually “deviant” stimulus elicits a mismatch negativity (MMN), reflecting preattentive detection of a change in acoustic pattern established by a repetitive stimulus train (Naatanen et al., 2007). MMN begins around the latency of N1 (100 ms) and peaks at 150–250 ms post-stimulus, indicating that the preattentive change detection mechanism operates at the level of auditory processing as indexed by N1-P2. On the other hand, the macaque counterpart of MMN has a peak latency of 80–90 ms (Javitt et al., 2000; Honing et al., 2012; Gil-da-Costa et al., 2013), commencing around mN1 (50 ms) and overlapping mP2 (70 ms), which is clearly earlier than mN2. Thus, it is reasonable to assume that N1 and mN1 reflect functionally comparable stages of auditory processing. This argument is further corroborated by an event-related potential component called object-related negativity (ORN), which reflects segregation of concurrent auditory objects, as it overlaps N1-P2 in humans (Alain et al., 2002; Alain and McDonald, 2007) and mN1-mP2 in monkeys (Fishman et al., 2014).

A competing hypothesis is that the frontal mN2 or the central mN2, not mN1, represents the human homolog of N1. A rationale for this proposition is that they are all prominent vertex negativities at approximately 100 ms. However, this tenet has several limitations. First it does not take into account the accumulating observation that evoked responses in the macaque generally have shorter latencies than those in humans (Legatt et al., 1986; Woodman, 2012). Second, it is also incompatible with the fact that MMN and ORN in macaque are elicited before the two subcomponents of mN2. Third, intracranial recordings of AEP from the supragranular layer of macaque primary auditory cortex have identified a negative peak around 50–60 ms (Fishman et al., 2000a, 2014; Fishman and Steinschneider, 2012), which likely contributes to our scalp-recorded mN1.

However, setting putative homologies aside, it is important to note that the central mN2 has the same latency, polarity and time window of integration as the N1. This highlights the fact that there is clear evidence for extended temporal integration in the macaque that matches that observed in the human N1, albeit not in the presumed functional homolog of the N1, the mN1, but rather the central mN2 which emerges at a similar latency as the N1. Similar latencies may be more important than homology in this specific context, given that latency imposes a strict upper limit on the time window of integration of a component. However, it is also important to point out that the frontal mN2 which occurs at the same latency as the N1, seems to have a very short integration window. This presumed co-existence of short and long temporal integration windows for components at similarly long latencies establishes an overall different pattern of temporal integration between the two species.

In contrast to the present study, previous invasive recordings of macaque auditory cortical responses to sounds have identified a negative peak in the latency range of 70–80 ms as a putative homolog of the human N1; this macaque component has been labeled as N70 (Arezzo et al., 1975), N1 (Javitt et al., 2000), or N85 (Teichert, 2016). There are several possible reasons for the discrepancy. First, the N70 (Arezzo et al., 1975) was recorded using epidural electrodes placed on the lateral surface of the frontal lobe, which is consistent with a radially oriented source in the frontal cortex. On the other hand, our use of midline electrodes was suitable for recording neural activities that are generated by tangentially oriented sources on the superior plane of the temporal lobe, such as the human vertex N1. In fact, the same intracranial study (Arezzo et al., 1975) recorded two negative responses, N60 and N100, when depth electrodes were placed in the superior temporal gyrus. It is therefore possible that the N60 and N100 correspond to the mN1 and mN2, respectively. A problem with this explanation, however, is that (Arezzo et al., 1975) did not report these negativities as being evident at epidural electrodes that were placed close to the midline. In addition, the N1 (Javitt et al., 2000) and N85 (Teichert, 2016) were recorded using midline electrodes. Second, the macaque N1 (Javitt et al., 2000) was defined as the most negative peak in the time window of 40–120 ms, and this wide latency range would have included both the mN1 and mN2 without distinguishing them. Third, whereas all of the above studies, including ours, used electrodes placed at the earlobe or mastoid as the reference, the N85 (Teichert, 2016) was recorded using a reference placed at Oz. It is difficult to compare wave morphologies if different references were used. Nevertheless, the waveforms in (Javitt et al., 2000) also show a negative peak at Cz around 80 ms, which is similar in scalp distribution and latency to the N85 (Teichert, 2016).

The effect of sound duration on mN2 amplitude was different between the Fz and Cz electrodes. We took this finding to indicate that the mN2 had multiple generators which had different time windows of integration. The frontal subcomponent maximal at Fz was elicited by even the briefest sound, indicating a quite short time window. By contrast, the temporal integration window for the central mN2 was much longer, because it was estimated to be between 50 and 100 ms. Importantly, both of these time windows were shorter than the time window for the human N2, which was clearly longer than 100 ms.

There were several limitations in this study. First, although the sample size of four was large compared with the sample size of many macaque experiments, the number of subjects was nevertheless smaller in the animal study compared with the human study. This led to an unbalance in the statistical power for detecting effects in the *post-hoc* analyses (Table 1). Second, the lack of a CAEP amplitude modulation with sound duration is not necessarily evidence for an absence of temporal integration. It is possible that the macaque cortex integrates auditory information by mechanisms that are not captured by this effect. Third, and most critical, our analyses and interpretations of the CAEP findings are based on the putative homologies between the macaque and human CAEP components, which remains to be proven by future research. An alternative interpretation, which focuses on the absolute latency of the neural responses rather than on the homologies of the components, is that the auditory cortices of humans and macaques have a comparable time window of integration, as the negative peak at around 100 ms (i.e., the N1 in humans and the mN2 in macaques) was modulated similarly by sound duration in both species.

## Conclusion

In conclusion, this study provides novel evidence for a species difference in the time scale of auditory processing at late stages of auditory cortical processing as indexed by N1 and later components of CAEP. The time scale of auditory cortical processing affects many, if not all, aspects of auditory perception, such as loudness (Fastl and Zwicker, 2007), speech (Lerner et al., 2011, 2014), and music (Lalitte and Bigand, 2006; Farbood et al., 2015) perception. To speculate, the shorter time window of auditory integration in monkeys might be related to the limited complexity and flexibility of their vocalizations. The repertoire of non-human primate vocalization is limited compared to the human speech (Gustison et al., 2012), and it is difficult to train non-human primates to produce vocal sounds outside their innate repertoire (Hayes and Hayes, 1951; Kojima, 2003; Fischer, 2017). This has been ascribed to the lack of an essential anatomical feature of the vocal tract, namely, a decent of the larynx (Lieberman et al., 1969) [see also: (Fitch et al., 2016)], and/or the lack of brain mechanisms responsible for the intricate control of speech organs (Holstege and Subramanian, 2016; Pisanski et al., 2016; Belyk and Brown, 2017). Thus, it is not surprising if their auditory cortical functions are organized differently than in humans, who can produce an infinite number of speech sounds by chaining phonemes in time. Further studies are warranted to elucidate how the elongation of auditory processing time scale supported the human evolution of language and other auditory cognitive abilities.

## Data Availability

The raw data supporting the conclusions of this manuscript will be made available by the authors, without undue reservation, to any qualified researcher.

## Ethics Statement

This human study was carried out in accordance with the recommendations of the Ethical Guidelines for Medical and Health Research Involving Human Subjects (Ministry of Education, Culture, Sports, Science, and Technology; Ministry of Health, Labor, and Welfare) with written informed consent from all subjects. All subjects gave written informed consent in accordance with the Declaration of Helsinki. The protocol was approved by the Internal Review Board of the University of Niigata. The animal study was carried out in accordance with the recommendations of the Guide for the Care and Use of Laboratory Animals of the National Research Council (1996) and the Guide for Care and Use of Laboratory Primates of Kyoto University. The protocol was approved by the Animal Care and Use Committee of Kyoto University.

## Author Contributions

KI conceived and designed the study. KI, MN, NK, and KN conducted the experiments. KI analyzed the data. KI and TN wrote the draft of the manuscript. All authors contributed to manuscript revision.

## Conflict of Interest Statement

The authors declare that the research was conducted in the absence of any commercial or financial relationships that could be construed as a potential conflict of interest.
